# Supplementary material for: PrP turnover in vivo and the time to effect of prion disease therapeutics
Source: PLoS Pathog. 2026 May 26;22(5):e1014263. doi: 10.1371/journal.ppat.1014263 (PMC13221148; doi:10.1371/journal.ppat.1014263)
Supplement: S1 Fig — Genomic DNA from a ki817 mouse was subjected to targeted sequencing for 152 kb around the PRNP locus using custom baits (1) (Twist biosciences) and aligned to the human reference genome. GRCh38 coordinates are shown. The knock-in allele spans 306 bases upstream of the human transcription start site (TSS, located at GRCh38 chr20:4,686,456) to 1 base downstream of the human transcription end site (TES, located at GRCh38 chr20:4701588). (PDF) [file ppat.1014263.s001.pdf]

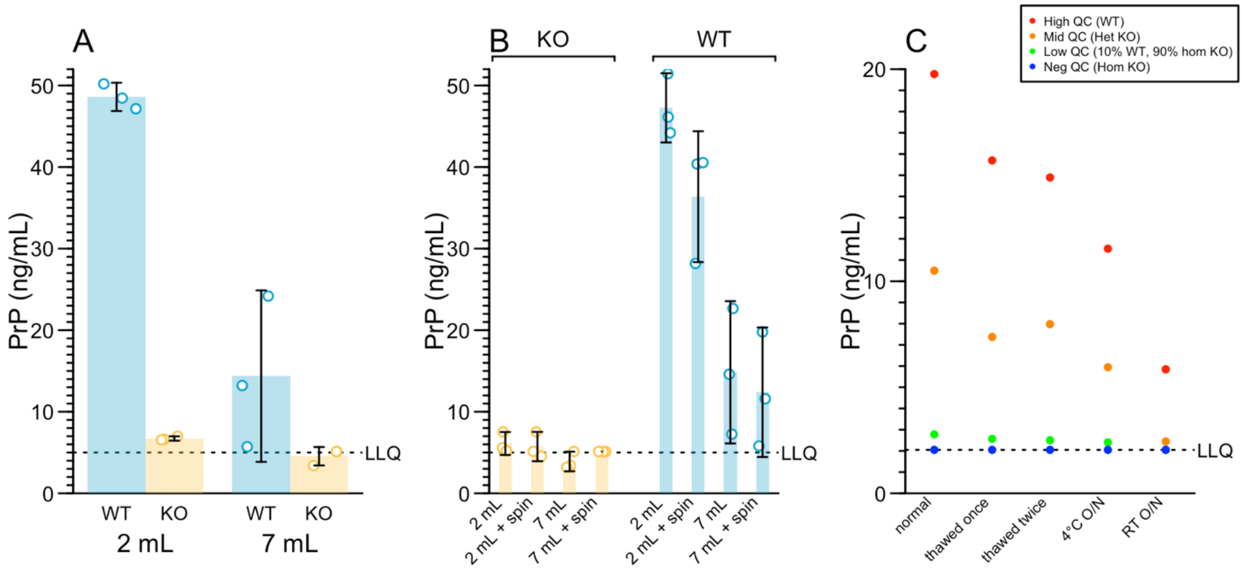

Figure S1. Assay development for ELISA quantification of PrP in colon. A) *Effect of homogenization tubes used. 7 mL tubes homogenize incompletely, leading to lower detection. These data used our standard ELISA conditions with 0.25 µg/mL detection antibody and were conducted at a 1:100 dilution.* ***B)*** *Centrifugation of samples does not rescue the under-recovery of PrP when 7 mL homogenization tubes are used.* ***C)*** *Stability study. PrP in colon samples is subject to loss upon freeze/thaw, time at 4°C or time at room temperature (RT) overnight (O/N). For this stability experiment we treated the lowest standard curve point, 0.02 ng/mL, as the lower limit of quantification (LLQ).*
