# Supplementary material for: PrP turnover in vivo and the time to effect of prion disease therapeutics
Source: PLoS Pathog. 2026 May 26;22(5):e1014263. doi: 10.1371/journal.ppat.1014263 (PMC13221148; doi:10.1371/journal.ppat.1014263)
Supplement: S2 Fig — A) The Charles River Labs (CRL) assay for VVEQ. Brain averages 4.7 times higher than colon. B) IQ Proteomics assay for VVEQ and GENF. Brain detection is 19.3 – 231 higher than colon, at odds with our Western and ELISA analysis (Fig 1) and the CRL assay (S1A Fig). Protein in colon may have been under-recovered due to incomplete homogenization. Nonetheless, this does not affect the proportion labeled measurement used in Fig 2. (PDF) [file ppat.1014263.s002.pdf]

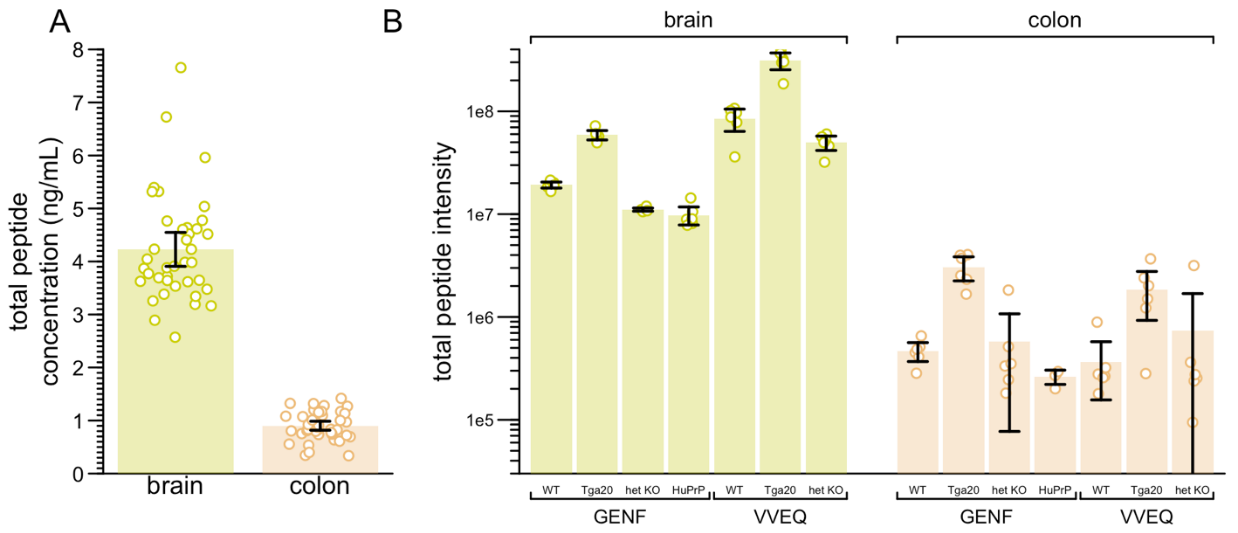

Figure S2. Total abundance of light+heavy peptide in the mass spectrometry assays. A) *The Charles River Labs (CRL) assay for VVEQ. Brain averages 4.7 times higher than colon.* ***B)*** *IQ Proteomics assay for VVEQ and GENF. Brain detection is 19.3 – 231 higher than colon, at odds with our Western and ELISA analysis (Figure 1) and the CRL assay (Figure S1A). Protein in colon may have been under-recovered due to incomplete homogenization. Nonetheless, this does not affect the proportion labeled measurement used in Figure 2.*
