# Supplementary material for: PrP turnover in vivo and the time to effect of prion disease therapeutics
Source: PLoS Pathog. 2026 May 26;22(5):e1014263. doi: 10.1371/journal.ppat.1014263 (PMC13221148; doi:10.1371/journal.ppat.1014263)
Supplement: S3 Fig — Each panel represents 1 of 19 peptides, whose identity and gene are shown at top. Synthetic peptides were serially diluted in triplicate. The upper section of each panel shows the coefficient of variation (%CV; y axis) among technical replicates at each dilution versus the nominal amount of peptide in amol (attomoles; x axis shared with lower section). The lower section shows the intensity (y axis) versus nominal amount. We defined each peptide’s LLQ (red dashed line) as the most dilute concentration for which the %CV of all concentrations equal or stronger than is ≤ 20%. Also shown are the mean heavy peptide intensities (blue dashed lines) for wild-type mouse brain and colon at day 8. Thus, if a blue line is above the red line, this means that in wild-type mice after 8 days, the heavy peptide in that tissue is detected above the LLQ. (PDF) [file ppat.1014263.s003.pdf]

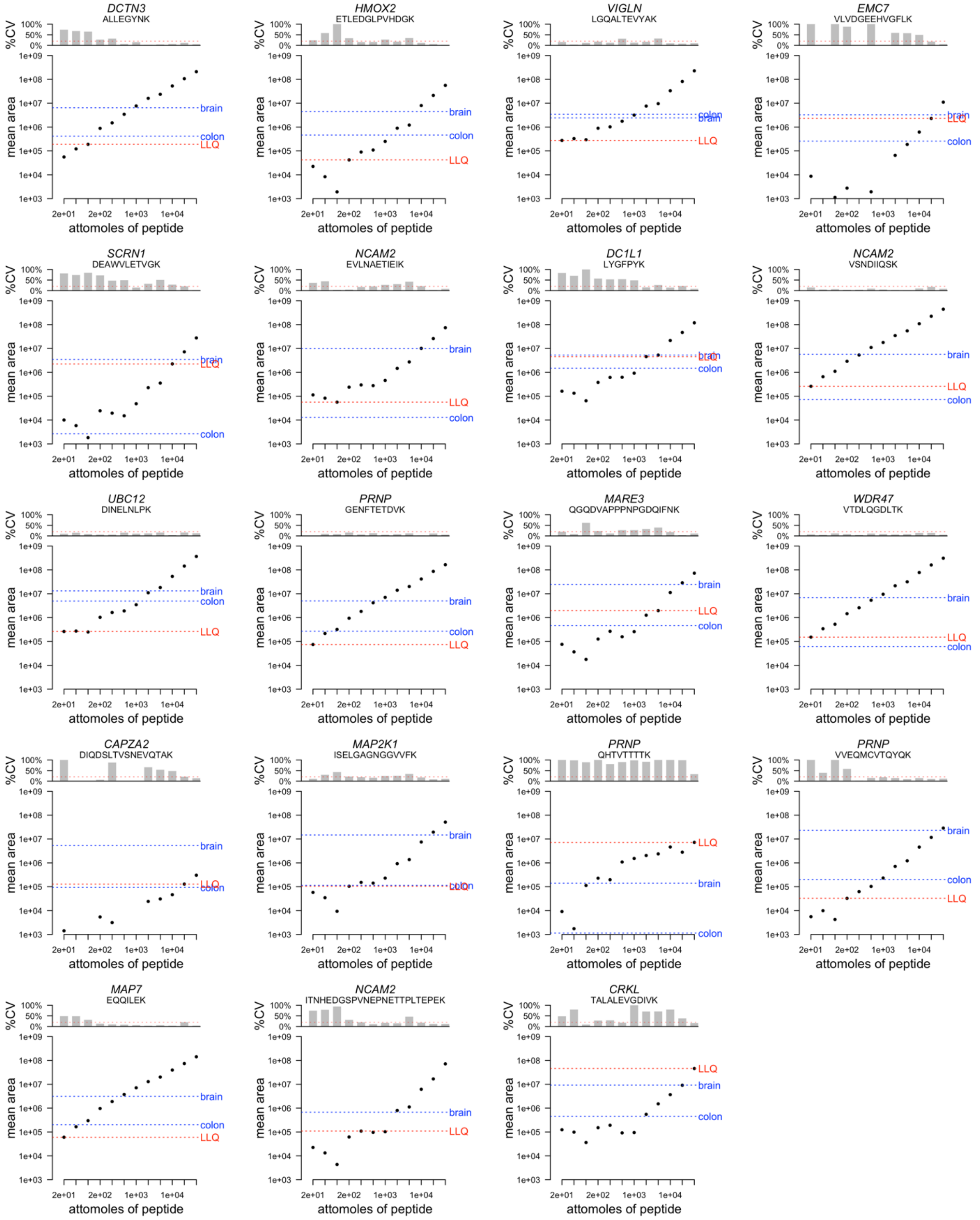

Figure S3. Quality control and LLQ determination for the IQ Proteomics assay. *Each panel represents 1 of 19 peptides, whose identity and gene are shown at top. Synthetic peptides were serially diluted in triplicate. The upper section of each panel shows the coefficient of variation (%CV; y axis) among technical replicates at each dilution versus the nominal amount of peptide*

in amol (attomoles; (x axis shared with lower section). The lower section shows the intensity (y axis) versus nominal amount. We defined each peptide's LLQ (red dashed line) as the most dilute concentration for which the %CV of all concentrations equal or stronger than is ≤20%. Also shown are the mean heavy peptide intensities (blue dashed lines) for wild-type mouse brain and colon at day 8. Thus, if a blue line is above the red line, this means that in wild-type mice after 8 days, the heavy peptide in that tissue is detected above the LLQ.
