# Supplementary material for: PrP turnover in vivo and the time to effect of prion disease therapeutics
Source: PLoS Pathog. 2026 May 26;22(5):e1014263. doi: 10.1371/journal.ppat.1014263 (PMC13221148; doi:10.1371/journal.ppat.1014263)
Supplement: S4 Fig — Genomic DNA from a ki817 mouse was subjected to targeted sequencing for 152 kb around the PRNP locus using custom baits (1) (Twist biosciences) and aligned to the human reference genome. GRCh38 coordinates are shown. The knock-in allele spans 306 bases upstream of the human transcription start site (TSS, located at GRCh38 chr20:4,686,456) to 1 base downstream of the human transcription end site (TES, located at GRCh38 chr20:4701588). (PDF) [file ppat.1014263.s004.pdf]

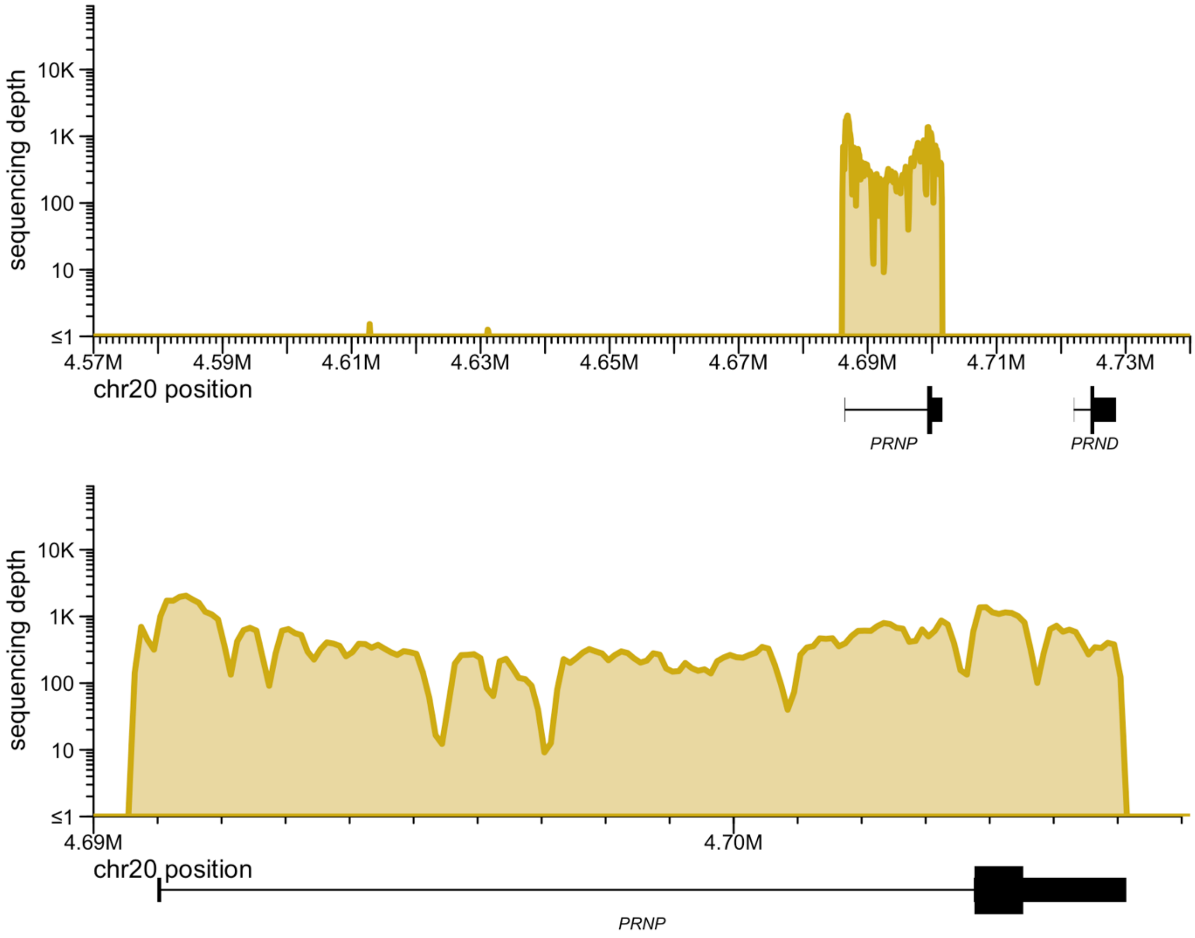

Figure S4. Human genomic sequence in ki817 mice. *Genomic DNA from a ki817 mouse was subjected to targeted sequencing for 152 kb around the PRNP locus using custom baits* (1) *(Twist biosciences) and aligned to the human reference genome. GRCh38 coordinates are shown. The knock-in allele spans 306 bases upstream of the human transcription start site (TSS, located at GRCh38 chr20:4,686,456) to 1 base downstream of the human transcription end site (TES, located at GRCh38 chr20:4701588).*

Sequencing reads demonstrating the breakpoints:

GRCm39 chr2:131751847 (TSS +0 bp) / human GRCh38 chr20:4686151 (TSS -306bp bases upstream of the TSS):

GGCGCGGCCATTGGTGAGCATCACGCCCCGCCCCTCGCCCAGCCTAGCTCCCGCCTGCC
CCGATTAAAGATGATTTTTACAGTCAATGAGCCACGTCAGGGAGCGATGGCACCCGCAGG
CGGTATCAACTGATGCAAGTGTTCAAG

human GRCh38 chr20:4701589 (1 base past the TES) / GRCm39 chr2:131780358 (1 base past TES)

TGAAATTAAACGAGCGAAGATGAGCACCACGGGGTTTGTTCTCTCTCCAATGCTCCGAGTC
CACTGTTTATCGCCAGGGTGGCTTGGGCTCATTTCACATCCCTGTCCCTGAGGGGCCTCG
GGTCTTACCTCTGGTCCTGTCTTGT
