# Supplementary material for: PrP turnover in vivo and the time to effect of prion disease therapeutics
Source: PLoS Pathog. 2026 May 26;22(5):e1014263. doi: 10.1371/journal.ppat.1014263 (PMC13221148; doi:10.1371/journal.ppat.1014263)
Supplement: S5 Fig — PrP ELISA on whole brain hemisphere homogenates from wild-type C57BL/6N mice or mice hemizygous for the Tga20 transgene array, on a background of endogenous PrP knockout (Tga20/0; ZH3/ZH3). The mean Tga20 value is 2.4x the wild-type result. The original report of the generation of the Tga20 line estimated that animals hemizygous for this transgene array expressed 6-7x wild-type PrP levels, by Western blot densitometry. We have shown that our ELISA provides quantitative estimates of relative PrP expression when samples are analyzed at the same dilution (2), and brain samples from all animals in this experiment were analyzed at the same 1:200 weight/vol final dilution. Transgene mapping of the Tga20 transgene array revealed integration at position (GRCm38) chr17:46,761,775–46,762,856, within intron 1 of Ptcra. The full transgene mapping report is provided in the study’s online git repository. (PDF) [file ppat.1014263.s005.pdf]

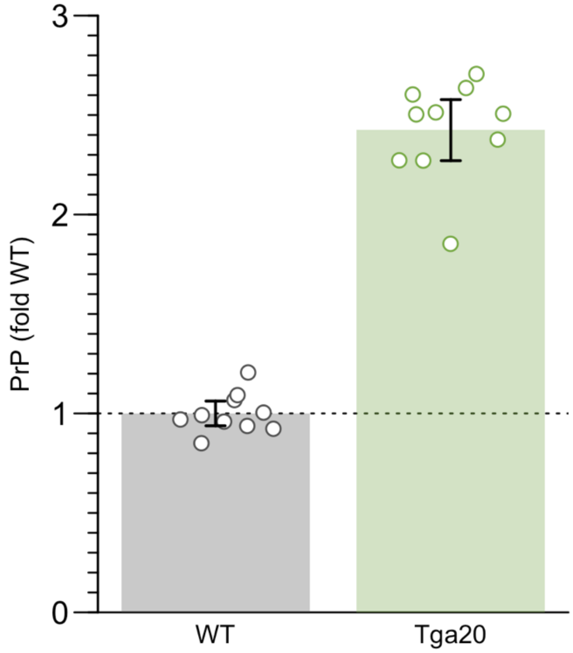

Figure S5. PrP expression in Tga20 mice. *PrP ELISA on whole brain hemisphere homogenates from wild-type C57BL/6N mice or mice hemizygous for the Tga20 transgene array, on a background of endogenous PrP knockout (Tga20/0; ZH3/ZH3). The mean Tga20 value is 2.4x the wild-type result. The original report of the generation of the Tga20 line estimated that animals hemizygous for this transgene array expressed 6-7x wild-type PrP levels, by Western blot densitometry. We have shown that our ELISA provides quantitative estimates of relative PrP expression when samples are analyzed at the same dilution (2), and brain samples from all animals in this experiment were analyzed at the same 1:200 weight/vol final dilution. Transgene mapping of the Tga20 transgene array revealed integration at position (GRCm38) chr17:46,761,775-46,762,856, within intron 1 of Ptcra. The full transgene mapping report is provided in the study's online git repository. The genomic breakpoints were identified as follows:*

5' integration site: GRCm38 chr17:46,761,775 (tail) fused to TG (homologous to chr2:131,909,936 (head))
ATCCCAGCGCCTACACACCCAACACTTCAATCTGTAATGAAATCCTATGCCCTCGTCTAGT
GTGTCTGAAGACACACTCCCGGCTCCCCCGCGTTGTCGGATCAGCAGACCGATTCTGGGC
GCTGCGTCGCATCGGTGGCAGGTAAGCG

3' integration site: TG (homologous to chr2:131,903,786 (head)) fused to GRCm38 chr17:46,762,856 (head) with 3 inserted bases
CTTGTTGGAAGAAGTGTGTAATTGGGGGTGAGCTTTGAAGTTTCAAATGCTTAAGCCAGGC
CCAGTATCACCCTCTCTGCCTTTTTGCTGCCTTTGGATCCGTCGCTTTGGAAATAATCTTTC
TTTTTTTTAAGATTTATTTTATGTAT
