# Supplementary material for: PrP turnover in vivo and the time to effect of prion disease therapeutics
Source: PLoS Pathog. 2026 May 26;22(5):e1014263. doi: 10.1371/journal.ppat.1014263 (PMC13221148; doi:10.1371/journal.ppat.1014263)
Supplement: S6 Fig — The empirically determined relative isotope abundance (RIA) calculated from N = 833 light-heavy to heavy-heavy double-lysine peptides from N = 596 proteins in paired brain and colon samples from N = 3 wild-type C57BL/6N animals after 8 days of labeled chow. A) Point estimates of RIA grouped by animal and tissue. Each point is a peptide. Segments and error bars represent 95% confidence intervals. B) Point estimates of RIA grouped by log10 bins of intensity (peptide abundance) and tissue. Each point is a peptide-animal tuple. Segments and error bars represent 95% confidence intervals. The source data, including exact number of peptides per bin, are provided in the S1 Data. (PDF) [file ppat.1014263.s006.pdf]

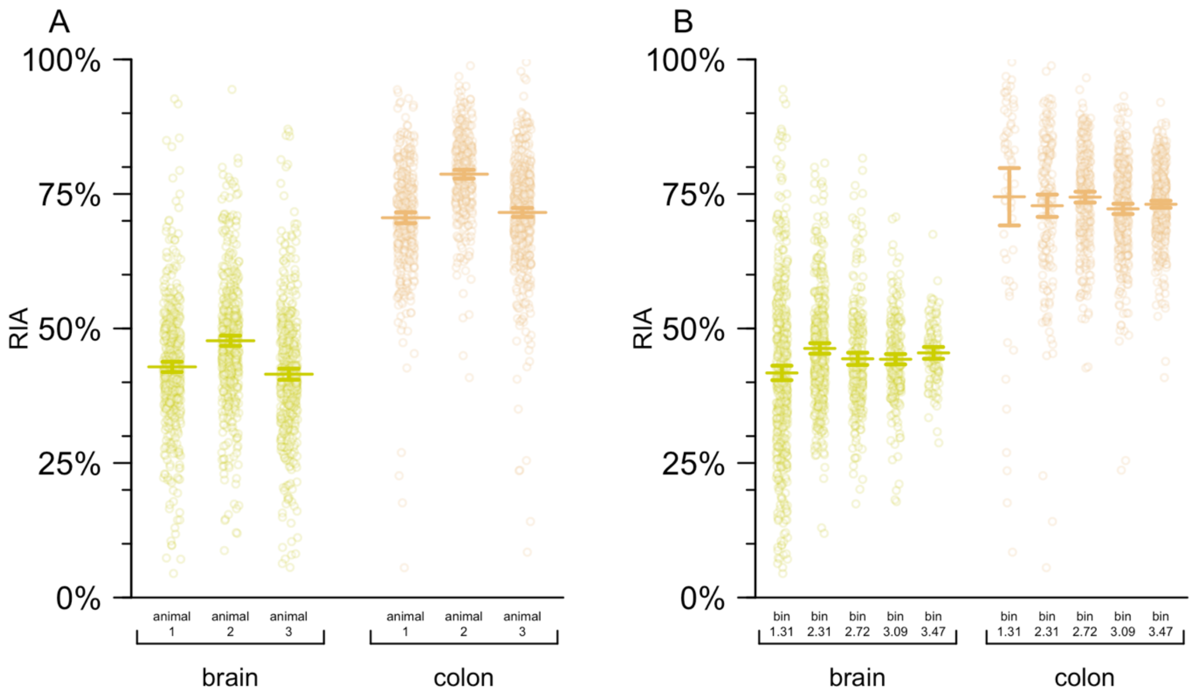

Figure S6. Empirical determination of heavy lysine availability in brain and colon. *The empirically determined relative isotope abundance (RIA) calculated from N=833 light-heavy to heavy-heavy double-lysine peptides from N=596 proteins in paired brain and colon samples from N=3 wild-type C57BL/6N animals after 8 days of labeled chow.* ***A)*** *Point estimates of RIA grouped by animal and tissue. Each point is a peptide. Segments and error bars represent 95% confidence intervals.* ***B)*** *Point estimates of RIA grouped by log10 bins of intensity (peptide abundance) and tissue. Each point is a peptide-animal tuple. Segments and error bars represent 95% confidence intervals. The source data, including exact number of peptides per bin, are provided in the Supplementary Data.*
