# Supplementary material for: PrP turnover in vivo and the time to effect of prion disease therapeutics
Source: PLoS Pathog. 2026 May 26;22(5):e1014263. doi: 10.1371/journal.ppat.1014263 (PMC13221148; doi:10.1371/journal.ppat.1014263)
Supplement: S7 Fig — Drug concentration (μg/g, y axis) versus days post-dose (x axis). Points represent individual animals (the same whole hemispheres used for qPCR), line segments represent means, and error bars represent 95% confidence intervals. (PDF) [file ppat.1014263.s007.pdf]

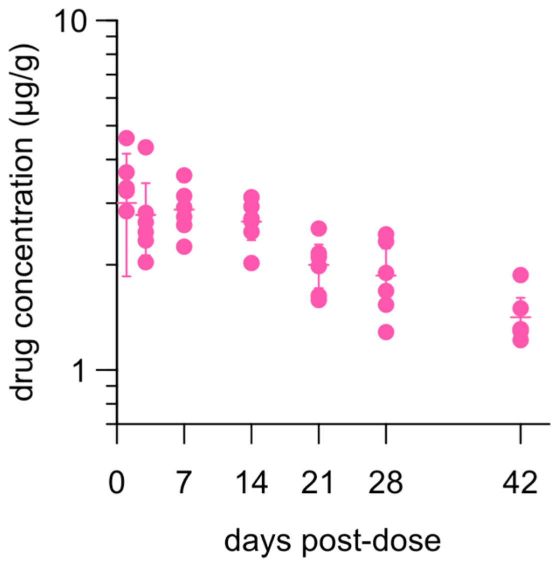

Figure S7. Pharmacokinetic (PK) parameters of ASO N in ki817 mice. *Drug concentration (µg/g, y axis) versus days post-dose (x axis). Points represent individual animals (the same whole hemispheres used for qPCR), line segments represent means, and error bars represent 95% confidence intervals.*
