# Supplementary material for: PrP turnover in vivo and the time to effect of prion disease therapeutics
Source: PLoS Pathog. 2026 May 26;22(5):e1014263. doi: 10.1371/journal.ppat.1014263 (PMC13221148; doi:10.1371/journal.ppat.1014263)
Supplement: S8 Fig — Shown on the y axis are total intensity (heavy + light peptide), grouped by genotype on the x axis; each point is one animal, bars represent means and error bars represent 95% confidence intervals. Age of animals and PrP peptide being monitored are indicated at top of each panel. (PDF) [file ppat.1014263.s008.pdf]

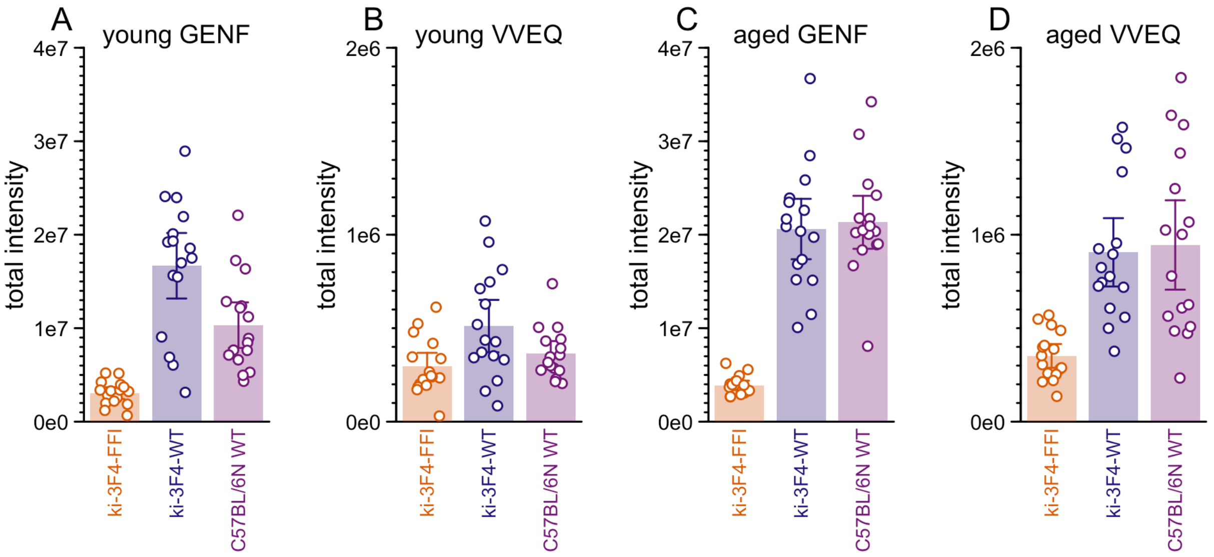

Figure S8. PrP peptide abundance by genotype and age in ki-3F4-FFI mice and controls. *Shown on the y axis are total intensity (heavy + light peptide), grouped by genotype on the x axis; each point is one animal, bars represent means and error bars represent 95% confidence intervals. Age of animals and PrP peptide being monitored are indicated at top of each panel.*
