# Supplementary material for: PrP turnover in vivo and the time to effect of prion disease therapeutics
Source: PLoS Pathog. 2026 May 26;22(5):e1014263. doi: 10.1371/journal.ppat.1014263 (PMC13221148; doi:10.1371/journal.ppat.1014263)
Supplement: S9 Fig — Single-rate (dashed line) and mixture-of-rates (solid line) models are shown. (PDF) [file ppat.1014263.s009.pdf]

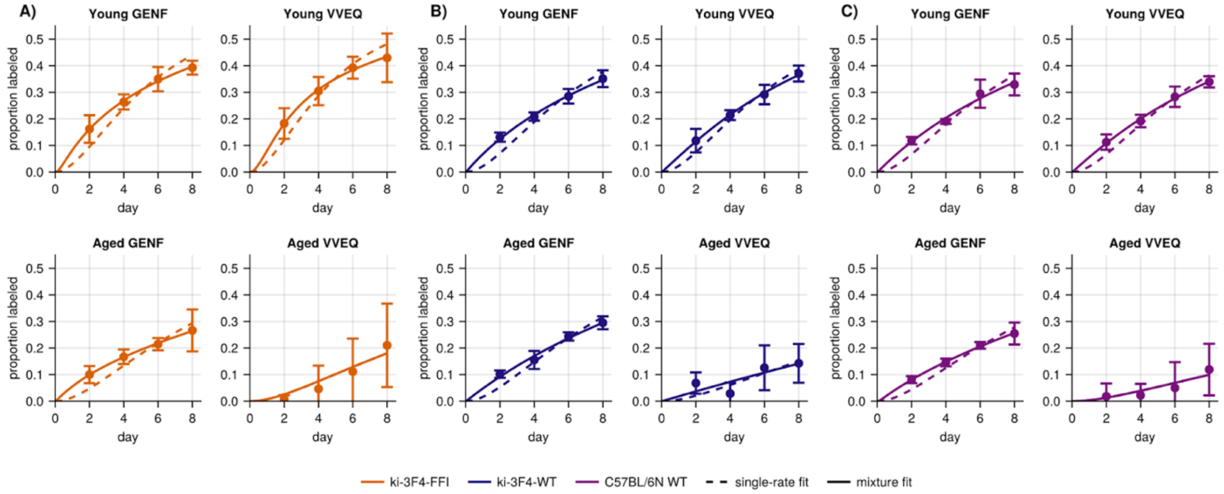

Figure S9. Comparison of single-rate and mixture-of-rates decay models. *For each peptide-age combination the genotypes A) ki-3F4-FFI B) ki-3F4-WT and C) C57BL/6N WT have the label accumulation plotted. Single-rate (dashed line) and mixture-of-rates (solid line) models are shown.*
