# Supplementary material for: PrP turnover in vivo and the time to effect of prion disease therapeutics
Source: PLoS Pathog. 2026 May 26;22(5):e1014263. doi: 10.1371/journal.ppat.1014263 (PMC13221148; doi:10.1371/journal.ppat.1014263)
Supplement: S1 Methods — (PDF) [file ppat.1014263.s010.pdf]

Supplementary Methods

Targeted mass spectrometry at IQ Proteomics.

Lysis performed at IQ Proteomics. 1mL of lysis buffer containing 8M Urea, 100 mM EPPS pH 8.5, and 1X HALT protease/phosphatase inhibitor (Pierce) was added to each sample. For complete lysis and shearing of genomic DNA, ceramic beads were added to each sample, and the samples were subjected to bead beating (Precellys Evolution) using four cycles at 5800 RPM for 30 seconds, followed by 30 second pause, with active cooling via chilled air circulation to 4°C. SDS was added to 1% w/w, and the lysates were cleared by centrifugation (2 minutes, 1000 x G, 4°C), followed by spin filtration, with an initial pass through 1.2 µM filter (Acroprep Advance, No. 2022-05, PALL Corporation) followed by a second pass through 0.2 µM filter (Acroprep Advance, No. 2022-15, PALL Corporation), after which 6 µL of lysate was removed from each sample into a 96-well plate for the purposes of total protein determination by BCA assay. The remainder of each sample was frozen at -80°C.

Protein Assay. From the reserved 6 µL, each lysate was evaluated via BCA assay (Pierce) according to the manufacturer's protocol. Triplicates at a 1:100 dilution and duplicates at a 1:200 dilution in PBS were incubated with 1X BCA reagent for one hour at 37°C. The samples were assayed for total protein concentration via absorbance at 562 nM in comparison to the standard curve comprised of serially diluted BSA.

Peptide Preparation. 50 µg of total protein (based on BCA assay) was removed from each sample to a new 96-well plate for subsequent processing. Sample volumes were normalized to 50 µL through the addition of the appropriate volume of lysis buffer. DTT was added to 5 mM final concentration in each sample, and samples were incubated at 37°C for 30 minutes. After equilibration to room temperature, iodoacetamide was added to 30 mM final concentration, and the samples were incubated at room temperature (RT) in the dark for 1 hour. Cysteine alkylation was quenched by addition of DTT to a final concentration of 25mM, and the samples were incubated in the dark for an additional 30 minutes at RT.

Digestion. Total protein was isolated via bead-based (SP3) precipitation protocol (3). Briefly, the magnetic beads were prepared by combining E3 and E7 Sera-Mag Carboxylate-Modified Magnetic SpeedBeads (Cytiva) at 1:1; vol:vol. Beads were washed three times with HPLC water prior to resuspension in lysis buffer for a final bead slurry concentration of 50 µg/µL. 15 µL of the pre-washed bead slurry and 100 µL 100% ethanol were added to each 50 µL sample containing 50 µg of protein. Proteins were allowed to bind and incubate with the bead slurry for 15 min at 25°C with gentle end-over-end rotation. Following protein binding, the supernatant was discarded, and the bead-captured proteins were washed three times with 80% ethanol and air dried. Purified protein-captured beads were resuspended in 100 mM EPPS, pH 8.0. Approximately 50 µg of protein/sample was digested at 25°C for 12 hours with lysyl endopeptidase (LysC, Wako Chemicals USA) at a 1:15; protease:protein (w/w) ratio. Following LysC digestion, the peptides were digested with trypsin at 37°C for 8 hours (Promega) at a 1:25; protease:protein (w/w) ratio. The digested peptides (supernatant) were purified and separated from the magnetic beads. Three post-digestion washes were performed to wash and ensure

elution of all peptides from the beads with the following buffers: 0.2% formic acid, 80% acetonitrile/0.1% formic acid, and 95% acetonitrile/0.1% formic acid. With every wash and elution, the supernatant was transferred and combined with the initial supernatant and dried to completion by vacuum centrifugation. Digested samples were desalted via C18 microextraction (Waters, Product Code 186002318), and peptide eluates were dried via speedvac. Desalted peptides were resuspended in 10uL of 0.2% formic acid for LC-MS analysis.

Preparation of potentially prion-infected for mass spectrometry. For ki-3F4-FFI mice and matched controls, as a precaution in case spontaneously formed prions were present in the brain samples, lysis and sample processing were performed in the prion laboratory at McLaughlin Research Institute and only denatured peptide samples were sent for proteomics, consistent with best practices reported elsewhere (4, 5). Whole brain hemispheres were homogenized at 10% wt/vol in PBS (Gibco 14190) with 2% sodium dodecyl sulfate. A 25 µL aliquot, corresponding to 2.5 mg of tissue or an estimated ~250 µg of input protein, was used for peptide isolation. 25 µL PBS (Gibco 14190) was added, followed by TCEP (Thermo Fisher 77720) to 20 mM final, the solution was incubated at 37°C for 30 minutes, cooled to RT, then iodoacetamide (Sigma A3221) was added to 15 mM final, then incubated at room temperature 60 minutes. TCEP was added again to 40 mM final. Methanol/chloroform precipitation was performed with 100 µL chloroform, 400 µL methanol, and 300 µL water, and centrifugatoion at 3,214 x G for 10 minutes, followed by 2 washes in 200 µL methanol. The protein pellet was air-dried, 10 µL of guanidine hydrochloride was added, transferred to a new microcentrifuge tube, and incubated at 37°C for 30 minutes. 75 µL of 0.2M EPPS pH 8.1 (VWR AAJ612960AE) and 75 µL of HPLC water were added, followed by 5 µg of LysC (Promega V1671), then overnight incubation at room temperature, then 5 µg of trypsin (Promega V5113), and incubation at 37°C for 6 hours. Resulting peptides were frozen at -80°C before advancing to post-digestion wash and desalting steps described above.

LC-MS Data Acquisition. All data were acquired using an Orbitrap Eclipse mass spectrometer equipped with FAIMS-Pro, operating in nano-flow mode using a EASY nanoLC-1200 (ThermoFisher) system (Thermo). Separation was achieved using an in-house packed C18 Column (Sepax GP-C18 resin) that was 30 cm in length with 75 µm inner diameter. Peptides were separated over a gradient composed of Buffer B (80% acetonitrile/0.2% formic acid) in Buffer A (0.2% formic acid) was run according to the following table:

Table A. LC protocol used at IQ Proteomics.

Time	duration (min)	flow (nL/min)	%B
0	0	300	10
50	50	300	40
55	5	300	100
60	5	300	100

Data was acquired via custom targeted assay with the instrument operating in tMS2 mode. Precursors were isolated using the quadrupole, and fragmented via HCD fragmentation. MS2

spectra were collected in the Orbitrap at 30K resolution. Maximum allowable injection time was set to 300ms.

Table B. Precursors in the IQ Proteomics assay.

m/z	z	RT Time (min)	Window (min)	HCD Collision Energy (%)	FAIMS CV (V)
874.4391	2	27.5	4	26	-40
877.4491	2	27.5	4	26	-40
614.861	2	41	4	20	-40
617.8711	2	41	4	20	-40
444.2367	2	30	4	20	-55
447.2579	2	30	4	20	-55
454.2478	2	17	4	20	-50
457.2579	2	17	4	20	-50
721.3879	2	30	4	29	-40
724.398	2	30	4	29	-40
705.349	2	20	4	26	-40
708.3591	2	20	4	26	-40
674.367	2	30	4	23	-40
677.377	2	30	4	23	-40
444.2453	2	11	4	17	-45
447.2553	2	11	4	17	-45
961.4738	2	29	4	26	-35
964.4838	2	29	4	26	-35
629.8481	2	30	4	20	-50
632.8582	2	30	4	20	-50
883.4156	3	20	4	29	-55
885.4223	3	20	4	29	-55
502.2746	2	13	4	20	-55
505.2846	2	13	4	20	-55
756.8629	2	19	4	23	-45
759.873	2	19	4	23	-45
570.2644	2	14	4	23	-50
573.2745	2	14	4	23	-50
508.7722	2	5	8	23	-65
511.7823	2	5	8	23	-65
623.8193	2	39	4	26	-50
626.8294	2	30	4	26	-50
528.2902	2	29	4	20	-50

531.3003	2	29	4	20	-50
599.8423	2	25	4	20	-40
596.8322	2	25	4	20	-40
548.303	2	18	4	20	-50
545.293	2	18	4	20	-50
705.2925	2	31	6	23	-45

LC-MS Data Analysis. LC-MS data was analyzed with the Skyline software package (www.skyline.ms). Peptide abundance was calculated as the ratio between the total area under the curve for the endogenous (light), chow-labeled ($^{13}C_6$ heavy) peptides. The following list of transitions was used:

Table C. Transitions used in the IQ Proteomics assay.

Protein Name	Peptide Sequence	Precursor Mz	Precursor Charge	Product Mz	Product Charge	Fragment Ion	Isotope Label Type
CAPZA2	DIQDSLTVSNEVQTAK	874.439063	2	1189.64229	1	y11	light
CAPZA2	DIQDSLTVSNEVQTAK	874.439063	2	1076.55823	1	y10	light
CAPZA2	DIQDSLTVSNEVQTAK	874.439063	2	975.510551	1	y9	light
CAPZA2	DIQDSLTVSNEVQTAK	874.439063	2	876.442137	1	y8	light
CAPZA2	DIQDSLTVSNEVQTAK	877.449127	2	1195.66242	1	y11	heavy
CAPZA2	DIQDSLTVSNEVQTAK	877.449127	2	1082.57836	1	y10	heavy
CAPZA2	DIQDSLTVSNEVQTAK	877.449127	2	981.53068	1	y9	heavy
CAPZA2	DIQDSLTVSNEVQTAK	877.449127	2	882.462266	1	y8	heavy
CRKL	TALALEVGDIVK	614.861003	2	943.545873	1	y9	light
CRKL	TALALEVGDIVK	614.861003	2	872.50876	1	y8	light
CRKL	TALALEVGDIVK	614.861003	2	759.424696	1	y7	light
CRKL	TALALEVGDIVK	614.861003	2	528.818607	2	y10	light
CRKL	TALALEVGDIVK	614.861003	2	286.176132	1	b3	light
CRKL	TALALEVGDIVK	617.871067	2	949.566002	1	y9	heavy
CRKL	TALALEVGDIVK	617.871067	2	878.528889	1	y8	heavy
CRKL	TALALEVGDIVK	617.871067	2	765.444825	1	y7	heavy
CRKL	TALALEVGDIVK	617.871067	2	531.828671	2	y10	heavy
CRKL	TALALEVGDIVK	617.871067	2	286.176132	1	b3	heavy
DC1L1	LYGFPYK	444.236721	2	774.382102	1	y6	light
DC1L1	LYGFPYK	444.236721	2	407.228896	1	y3	light
DC1L1	LYGFPYK	447.246786	2	780.402231	1	y6	heavy
DC1L1	LYGFPYK	447.246786	2	413.249025	1	y3	heavy
DCTN3	ALLEGYNK	454.247817	2	723.367181	1	y6	light
DCTN3	ALLEGYNK	454.247817	2	610.283117	1	y5	light

DCTN3	ALLEGYNK	454.247817	2	481.240524	1	y4	light
DCTN3	ALLEGYNK	457.257882	2	729.38731	1	y6	heavy
DCTN3	ALLEGYNK	457.257882	2	616.303246	1	y5	heavy
DCTN3	ALLEGYNK	457.257882	2	487.260653	1	y4	heavy
EMC7	VLVDGEEHVGFLK	721.387916	2	1229.61608	1	y11	light
EMC7	VLVDGEEHVGFLK	721.387916	2	1130.54766	1	y10	light
EMC7	VLVDGEEHVGFLK	721.387916	2	1015.52072	1	y9	light
EMC7	VLVDGEEHVGFLK	721.387916	2	615.311677	2	y11	light
EMC7	VLVDGEEHVGFLK	721.387916	2	565.77747	2	y10	light
EMC7	VLVDGEEHVGFLK	724.397981	2	1235.63621	1	y11	heavy
EMC7	VLVDGEEHVGFLK	724.397981	2	1136.56779	1	y10	heavy
EMC7	VLVDGEEHVGFLK	724.397981	2	1021.54085	1	y9	heavy
EMC7	VLVDGEEHVGFLK	724.397981	2	618.321742	2	y11	heavy
EMC7	VLVDGEEHVGFLK	724.397981	2	568.787535	2	y10	heavy
HMOX2	ETLEDGLPVHDGK	705.348988	2	1066.51636	1	y10	light
HMOX2	ETLEDGLPVHDGK	705.348988	2	937.473771	1	y9	light
HMOX2	ETLEDGLPVHDGK	705.348988	2	822.446828	1	y8	light
HMOX2	ETLEDGLPVHDGK	705.348988	2	652.3413	1	y6	light
HMOX2	ETLEDGLPVHDGK	705.348988	2	456.220122	1	y4	light
HMOX2	ETLEDGLPVHDGK	708.359052	2	1072.53649	1	y10	heavy
HMOX2	ETLEDGLPVHDGK	708.359052	2	943.4939	1	y9	heavy
HMOX2	ETLEDGLPVHDGK	708.359052	2	828.466957	1	y8	heavy
HMOX2	ETLEDGLPVHDGK	708.359052	2	658.361429	1	y6	heavy
HMOX2	ETLEDGLPVHDGK	708.359052	2	462.240251	1	y4	heavy
MAP2K1	ISELGAGNGGVVFK	674.366984	2	1018.56801	1	y11	light
MAP2K1	ISELGAGNGGVVFK	674.366984	2	905.483942	1	y10	light
MAP2K1	ISELGAGNGGVVFK	674.366984	2	848.462478	1	y9	light
MAP2K1	ISELGAGNGGVVFK	674.366984	2	777.425364	1	y8	light
MAP2K1	ISELGAGNGGVVFK	677.377048	2	1024.58814	1	y11	heavy
MAP2K1	ISELGAGNGGVVFK	677.377048	2	911.504071	1	y10	heavy
MAP2K1	ISELGAGNGGVVFK	677.377048	2	854.482607	1	y9	heavy
MAP2K1	ISELGAGNGGVVFK	677.377048	2	783.445493	1	y8	heavy
MAP7	EQQILEK	444.245275	2	630.382102	1	y5	light
MAP7	EQQILEK	444.245275	2	502.323525	1	y4	light
MAP7	EQQILEK	444.245275	2	389.239461	1	y3	light
MAP7	EQQILEK	444.245275	2	315.694689	2	y5	light
MAP7	EQQILEK	444.245275	2	386.167024	1	b3	light
MAP7	EQQILEK	447.255339	2	636.402231	1	y5	heavy
MAP7	EQQILEK	447.255339	2	508.343654	1	y4	heavy

MAP7	EQQILEK	447.255339	2	395.25959	1	y3	heavy
MAP7	EQQILEK	447.255339	2	318.704754	2	y5	heavy
MAP7	EQQILEK	447.255339	2	386.167024	1	b3	heavy
MARE3	QGQDVAPPPNPGDQIFNK	961.473771	2	1394.70629	1	y13	light
MARE3	QGQDVAPPPNPGDQIFNK	961.473771	2	1323.66918	1	y12	light
MARE3	QGQDVAPPPNPGDQIFNK	961.473771	2	1226.61641	1	y11	light
MARE3	QGQDVAPPPNPGDQIFNK	961.473771	2	1129.56365	1	y10	light
MARE3	QGQDVAPPPNPGDQIFNK	961.473771	2	918.467957	1	y8	light
MARE3	QGQDVAPPPNPGDQIFNK	964.483836	2	1400.72642	1	y13	heavy
MARE3	QGQDVAPPPNPGDQIFNK	964.483836	2	1329.68931	1	y12	heavy
MARE3	QGQDVAPPPNPGDQIFNK	964.483836	2	1232.63654	1	y11	heavy
MARE3	QGQDVAPPPNPGDQIFNK	964.483836	2	1135.58378	1	y10	heavy
MARE3	QGQDVAPPPNPGDQIFNK	964.483836	2	924.488086	1	y8	heavy
NCAM2	EVLNAETIEIK	629.848092	2	1030.5779	1	y9	light
NCAM2	EVLNAETIEIK	629.848092	2	917.493838	1	y8	light
NCAM2	EVLNAETIEIK	629.848092	2	803.45091	1	y7	light
NCAM2	EVLNAETIEIK	629.848092	2	732.413797	1	y6	light
NCAM2	EVLNAETIEIK	632.858157	2	1036.59803	1	y9	heavy
NCAM2	EVLNAETIEIK	632.858157	2	923.513967	1	y8	heavy
NCAM2	EVLNAETIEIK	632.858157	2	809.471039	1	y7	heavy
NCAM2	EVLNAETIEIK	632.858157	2	738.433926	1	y6	heavy
NCAM2	VSNDIIQSK	502.274563	2	904.473437	1	y8	light
NCAM2	VSNDIIQSK	502.274563	2	817.441408	1	y7	light
NCAM2	VSNDIIQSK	502.274563	2	588.371538	1	y5	light
NCAM2	VSNDIIQSK	502.274563	2	475.287474	1	y4	light
NCAM2	VSNDIIQSK	505.284628	2	910.493566	1	y8	heavy
NCAM2	VSNDIIQSK	505.284628	2	823.461537	1	y7	heavy
NCAM2	VSNDIIQSK	505.284628	2	594.391667	1	y5	heavy
NCAM2	VSNDIIQSK	505.284628	2	481.307603	1	y4	heavy
PRNP	VVEQMC[+57]VTQYQK	756.862894	2	1057.48051	1	y8	light
PRNP	VVEQMC[+57]VTQYQK	756.862894	2	926.440028	1	y7	light
PRNP	VVEQMC[+57]VTQYQK	756.862894	2	667.340966	1	y5	light
PRNP	VVEQMC[+57]VTQYQK	759.872958	2	1063.50064	1	y8	heavy
PRNP	VVEQMC[+57]VTQYQK	759.872958	2	932.460157	1	y7	heavy
PRNP	VVEQMC[+57]VTQYQK	759.872958	2	673.361095	1	y5	heavy
PRNP	GENFTETDVK	570.264393	2	953.457452	1	y8	light
PRNP	GENFTETDVK	570.264393	2	839.414525	1	y7	light
PRNP	GENFTETDVK	570.264393	2	692.346111	1	y6	light
PRNP	GENFTETDVK	570.264393	2	591.298432	1	y5	light

PRNP	GENFTETDVK	573.274457	2	959.477581	1	y8	heavy
PRNP	GENFTETDVK	573.274457	2	845.434654	1	y7	heavy
PRNP	GENFTETDVK	573.274457	2	698.36624	1	y6	heavy
PRNP	GENFTETDVK	573.274457	2	597.318561	1	y5	heavy
PRNP	QHTVTTTTK	508.772188	2	751.41961	1	y7	light
PRNP	QHTVTTTTK	508.772188	2	650.371932	1	y6	light
PRNP	QHTVTTTTK	508.772188	2	450.255839	1	y4	light
PRNP	QHTVTTTTK	508.772188	2	349.208161	1	y3	light
PRNP	QHTVTTTTK	511.782252	2	757.439739	1	y7	heavy
PRNP	QHTVTTTTK	511.782252	2	656.392061	1	y6	heavy
PRNP	QHTVTTTTK	511.782252	2	456.275968	1	y4	heavy
PRNP	QHTVTTTTK	511.782252	2	355.22829	1	y3	heavy
SCRN1	DEAWVLETVGK	623.819335	2	1002.56186	1	y9	light
SCRN1	DEAWVLETVGK	623.819335	2	931.524744	1	y8	light
SCRN1	DEAWVLETVGK	623.819335	2	745.445431	1	y7	light
SCRN1	DEAWVLETVGK	623.819335	2	646.377017	1	y6	light
SCRN1	DEAWVLETVGK	623.819335	2	502.193239	1	b4	light
SCRN1	DEAWVLETVGK	626.8294	2	1008.58199	1	y9	heavy
SCRN1	DEAWVLETVGK	626.8294	2	937.544873	1	y8	heavy
SCRN1	DEAWVLETVGK	626.8294	2	751.46556	1	y7	heavy
SCRN1	DEAWVLETVGK	626.8294	2	652.397146	1	y6	heavy
SCRN1	DEAWVLETVGK	626.8294	2	502.193239	1	b4	heavy
UBC12	DINELNLPK	528.290213	2	827.462144	1	y7	light
UBC12	DINELNLPK	528.290213	2	713.419216	1	y6	light
UBC12	DINELNLPK	528.290213	2	584.376623	1	y5	light
UBC12	DINELNLPK	528.290213	2	471.292559	1	y4	light
UBC12	DINELNLPK	528.290213	2	414.23471	2	y7	light
UBC12	DINELNLPK	531.300278	2	833.482273	1	y7	heavy
UBC12	DINELNLPK	531.300278	2	719.439345	1	y6	heavy
UBC12	DINELNLPK	531.300278	2	590.396752	1	y5	heavy
UBC12	DINELNLPK	531.300278	2	477.312688	1	y4	heavy
UBC12	DINELNLPK	531.300278	2	417.244774	2	y7	heavy
VIGLN	LGQALTEVYAK	596.832245	2	1079.57315	1	y10	light
VIGLN	LGQALTEVYAK	596.832245	2	894.49311	1	y8	light
VIGLN	LGQALTEVYAK	596.832245	2	609.324253	1	y5	light
VIGLN	LGQALTEVYAK	596.832245	2	480.28166	1	y4	light
VIGLN	LGQALTEVYAK	596.832245	2	511.779482	2	y9	light
VIGLN	LGQALTEVYAK	599.84231	2	1085.59328	1	y10	heavy
VIGLN	LGQALTEVYAK	599.84231	2	900.513239	1	y8	heavy

VIGLN	LGQALTEVYAK	599.84231	2	615.344382	1	y5	heavy
VIGLN	LGQALTEVYAK	599.84231	2	486.301789	1	y4	heavy
VIGLN	LGQALTEVYAK	599.84231	2	514.789546	2	y9	heavy
WDR47	VTDLQGDLTK	545.292953	2	990.510216	1	y9	light
WDR47	VTDLQGDLTK	545.292953	2	661.351531	1	y6	light
WDR47	VTDLQGDLTK	545.292953	2	316.150312	1	b3	light
WDR47	VTDLQGDLTK	548.303018	2	996.530345	1	y9	heavy
WDR47	VTDLQGDLTK	548.303018	2	667.37166	1	y6	heavy
WDR47	VTDLQGDLTK	548.303018	2	316.150312	1	b3	heavy

Lower limit of quantification (LLQ) sample preparation. Internal standard peptides labeled with $^{13}C_6$ $^{15}N_2$ lysine (Biosynth, Gardner MA, USA) were mixed into an equimolar solution, and a 12-point, two-fold serial dilution of the peptide mix was prepared by diluting into 0.2% formic acid solution such that the following amounts of peptide were evaluated by LC-MS according to Table S4.

Table D. Synthetic peptide serial dilutions to determine lower limit of quantification (LLQ)

curve point	fmol peptide on column
12	40
11	20
10	10
9	5
8	2.5
7	1.25
6	0.625
5	0.313
4	0.156
3	0.0781
2	0.039
1	0.0195

LLQ LC-MS Runs. LC and MS instrument parameters for LoQ runs were identical to those for the study samples with the exception that masses corresponding to the heavy synthetic peptide standards (containing terminal $^{13}C_6$ $^{15}N_2$ lysine) were evaluated:

Table E. Precursors for LLQ determination.

m/z	z	RT Time (min)	Window (min)	HCD Collision Energy (%)	FAIMS CV (V)
874.4391	2	27.7	10	26	-40
878.4462	2	27.7	10	26	-40
614.861	2	40.2	10	20	-40
618.8681	2	40.2	10	20	-40
444.2367	2	31.6	10	20	-55
448.2438	2	31.6	10	20	-55
454.2478	2	20.3	10	20	-50
458.2549	2	20.3	10	20	-50
721.3879	2	30.9	10	29	-40
725.395	2	30.9	10	29	-40
705.349	2	22.1	10	26	-40
709.3561	2	22.1	10	26	-40
674.367	2	30.5	10	23	-40
678.3741	2	30.5	10	23	-40
444.2453	2	13.4	10	17	-45
448.2524	2	13.4	10	17	-45
961.4738	2	29.5	10	26	-35
965.4809	2	29.5	10	26	-35
629.8481	2	31	10	20	-50
633.8552	2	31	10	20	-50
883.4156	3	21.6	10	29	-55
886.087	3	21.6	10	29	-55
502.2746	2	15.2	10	20	-55
506.2817	2	15.2	10	20	-55
400.2554	2	30.7	10	20	-60
405.2596	2	30.7	10	20	-60
522.7409	2	10	10	20	-55
527.745	2	10	10	20	-55
778.3658	2	27.4	10	23	-40
783.3699	2	27.4	10	23	-40
544.7358	2	10.8	10	20	-60
549.7399	2	10.8	10	20	-60
756.8629	2	21.1	10	23	-45
760.87	2	21.1	10	23	-45
570.2644	2	16	10	23	-50

574.2715	2	16	10	23	-50
508.7722	2	7.5	10	23	-65
512.7793	2	7.5	10	23	-65
545.2572	2	8.8	10	32	-60
550.2614	2	8.8	10	32	-60
623.8193	2	37.9	10	26	-50
627.8264	2	37.9	10	26	-50
483.238	2	18	10	26	-50
488.2421	2	18	10	26	-50
525.8084	2	43.5	10	20	-55
530.8125	2	43.5	10	20	-55
528.2902	2	29.7	10	20	-50
532.2973	2	29.7	10	20	-50
596.8322	2	26.7	10	20	-40
600.8393	2	26.7	10	20	-40
545.293	2	19.7	10	20	-50
549.3001	2	19.7	10	20	-50

Table F. Fragment ions corresponding to the synthetic peptides for LLQ determination.

Peptide	Precursor m/z	Fragment m/z	Fragment Ion
DIQDSLTVSNEVQTAK	878.446162	1197.65649	y11
DIQDSLTVSNEVQTAK	878.446162	1084.57243	y10
DIQDSLTVSNEVQTAK	878.446162	983.52475	y9
DIQDSLTVSNEVQTAK	878.446162	884.456336	y8
TALALEVGDIVK	618.868102	951.560072	y9
TALALEVGDIVK	618.868102	880.522959	y8
TALALEVGDIVK	618.868102	767.438895	y7
TALALEVGDIVK	618.868102	532.825706	y10
LYGFPYK	448.243821	782.396301	y6
LYGFPYK	448.243821	619.332973	y5
LYGFPYK	448.243821	562.311509	y4
LYGFPYK	448.243821	415.243095	y3
LYGFPYK	448.243821	391.701789	y6
ALLEGYNK	458.254917	731.38138	y6
ALLEGYNK	458.254917	618.297316	y5
ALLEGYNK	458.254917	489.254723	y4
VLVDGEEHVGFLK	725.395016	1237.63028	y11

VLVDGEEHVGFLK	725.395016	1138.56186	y10
VLVDGEEHVGFLK	725.395016	1023.53492	y9
VLVDGEEHVGFLK	725.395016	619.318777	y11
VLVDGEEHVGFLK	725.395016	569.78457	y10
ETLEDGLPVHDGK	709.356087	1074.53056	y10
ETLEDGLPVHDGK	709.356087	945.48797	y9
ETLEDGLPVHDGK	709.356087	830.461027	y8
ETLEDGLPVHDGK	709.356087	660.355499	y6
ETLEDGLPVHDGK	709.356087	464.234321	y4
ISELGAGNGGVVFK	678.374083	1026.58221	y11
ISELGAGNGGVVFK	678.374083	913.498141	y10
ISELGAGNGGVVFK	678.374083	856.476677	y9
ISELGAGNGGVVFK	678.374083	785.439563	y8
EQQILEK	448.252374	638.396301	y5
EQQILEK	448.252374	510.337724	y4
EQQILEK	448.252374	397.25366	y3
EQQILEK	448.252374	319.701789	y5
EQQILEK	448.252374	386.167024	b3
QGQDVAPPPNPGDQIFNK	965.480871	1402.72049	y13
QGQDVAPPPNPGDQIFNK	965.480871	1331.68338	y12
QGQDVAPPPNPGDQIFNK	965.480871	1234.63061	y11
QGQDVAPPPNPGDQIFNK	965.480871	1137.57785	y10
QGQDVAPPPNPGDQIFNK	965.480871	926.482156	y8
EVLNAETIEIK	633.855192	1038.5921	y9
EVLNAETIEIK	633.855192	925.508037	y8
EVLNAETIEIK	633.855192	811.465109	y7
EVLNAETIEIK	633.855192	740.427996	y6
EVLNAETIEIK	633.855192	611.385402	y5
ITNHEDGSPVNEPNETTPLTEPEK	886.086987	1363.6831	y12
ITNHEDGSPVNEPNETTPLTEPEK	886.086987	821.449459	y7
ITNHEDGSPVNEPNETTPLTEPEK	886.086987	1293.57059	b12
VSNDIIQSK	506.281663	912.487636	y8
VSNDIIQSK	506.281663	825.455607	y7
VSNDIIQSK	506.281663	711.41268	y6
VSNDIIQSK	506.281663	596.385737	y5
VSNDIIQSK	506.281663	483.301673	y4
VVEQMC[+57.021464]VTQYQK	760.869993	1065.49471	y8
VVEQMC[+57.021464]VTQYQK	760.869993	934.454227	y7
VVEQMC[+57.021464]VTQYQK	760.869993	675.355165	y5

GENFTETDVK	574.271492	961.471651	y8
GENFTETDVK	574.271492	847.428724	y7
GENFTETDVK	574.271492	700.36031	y6
GENFTETDVK	574.271492	599.312631	y5
QHTVTTTTK	512.779287	759.433809	y7
QHTVTTTTK	512.779287	658.386131	y6
QHTVTTTTK	512.779287	559.317717	y5
QHTVTTTTK	512.779287	458.270038	y4
QHTVTTTTK	512.779287	357.22236	y3
DEAWVLETVGK	627.826435	1010.57606	y9
DEAWVLETVGK	627.826435	939.538943	y8
DEAWVLETVGK	627.826435	753.45963	y7
DEAWVLETVGK	627.826435	654.391216	y6
DEAWVLETVGK	627.826435	502.193239	b4
DINELNLPK	532.297313	835.476343	y7
DINELNLPK	532.297313	721.433415	y6
DINELNLPK	532.297313	592.390822	y5
DINELNLPK	532.297313	479.306758	y4
DINELNLPK	532.297313	418.241809	y7
LGQALTEVYAK	600.839345	1087.58735	y10
LGQALTEVYAK	600.839345	902.507309	y8
LGQALTEVYAK	600.839345	831.470195	y7
LGQALTEVYAK	600.839345	718.386131	y6
LGQALTEVYAK	600.839345	515.786581	y9
VTDLQGDLTK	549.300053	998.524415	y9
VTDLQGDLTK	549.300053	897.476737	y8
VTDLQGDLTK	549.300053	669.36573	y6
VTDLQGDLTK	549.300053	541.307152	y5
VTDLQGDLTK	549.300053	316.150312	b3

Targeted mass spectrometry at Charles River Labs.

Tissue homogenization. Colon and brain tissue were homogenized in TPER reagent (Thermo No. 78510) with 1X HALT protease inhibitor (Thermo No. 78430) at a ratio of 1 g tissue to 9 mL buffer.

Total protein estimation by BCA assay. 10 µL aliquots of tissue homogenates (see above) were diluted 10-fold in water for total protein estimation. Seven calibration standards concentrations (2, 1, 0.5, 0.25, 0.125, 0.0625, 0.03125) were prepared with serial 2x dilution in milliQ water. BCA reagent was prepared by mixing reagent A and B in a 50:1 ratio. 20 µL of sample and 200 µL of BCA reagent were added to each well of a clear bottom 96 well plate and incubated at 37°C for 20 minutes. Absorbance of each well was read at 562 nm using a UV spectrometer. Samples were diluted to 3 mg/mL total protein concentration based on BCA assay data.

Extraction procedure. 60 µL of mixed reagent consisting of 20 µL bovine serum albumin (BSA), 30 µL ammonium bicarbonate (ABC, 100 mM), and 10 µL dithiothreitol (DTT, 250 mM) was added to 80 µL of the 3 mg/mL sample and mixed before incubating at 95°C for 10 minutes at 300 rpm shaking. Samples were then allowed to cool for 10 minutes at room temperature. 10 µL of iodoacetamide (IAA, 500 mM) was added to the samples, vortexed to mix and incubated at RT in the dark for 30 minutes. 1 mL of cold acetone was added to the samples and vortexed to mix again prior to incubating at -80°C for 30 minutes. Samples were spun at 16,000 x G for 5 minutes and supernatant was discarded. The protein pellet was dried in the hood, washed with 500 µL cold methanol and dried for 30 additional minutes in the hood at room temperature. The protein pellet was resuspended in 60 µL of 50 mM ABC plus 20 µL of diluted trypsin (0.16 µg/µL) for a final quantity of 3.2 µg trypsin to 240 µg protein (1:75 enzyme:protein ratio) and mixed with a pipette before incubating for 16 hours at 37°C. An internal standard (IS) of peptide with isotopically labeled lysine (^{13}C, ^{15}N) custom synthesized by Vivitide was used. The reaction was stopped by adding 10 µL of IS (prepared in 0.5% FA ACN-H_2O 80:20) and 10 µL FA in ACN:H_2O (80:20) and vortexing to mix before centrifugation at 8,000 x G for 5 minutes. Supernatants were transferred to LCMS vials.

Calibration curve. 80 µL of 50 mM ammonium bicarbonate was aliquoted into the tubes designated for blanks and standards. The calibration curve was prepared with light peptides standard custom synthesized by Vivitide. 10 µL of ACN:H2O:FA 1:10:1 was aliquoted into each 80 µL sample, blank, or standard. 10 µL of standards were added to tubes labeled for standards. 10 µL of IS in 80:20:0.5 ACN:H_2O:FA was added to all standard, QC, and blank tubes except for double blank tubes. 10 µL of 80:20:0.5 ACN:H_2O:FA was added to double blanks. All Eppendorf tubes were covered and vortexed prior to being centrifuged for 5 minutes at 8000 x G. At least 80 µL of the samples were transferred into a clean 96-well plate.
